# When Every Minute Counts: Predicting Pre-Hospital Deliveries and Neonatal Risk in Emergency Medical Services Using Data-Driven Models

**DOI:** 10.3390/jcm15030941

**Published:** 2026-01-23

**Authors:** Joanna Wach, Łukasz Lewandowski, Jakub Staniczek, Michał Czapla

**Affiliations:** 1 Division of Specialist Care in Midwifery and Gynaecology, Department of Midwifery, Faculty of Nursing and Midwifery, Wroclaw Medical University, 51-618 Wroclaw, Poland; 2 Department of Medical Biochemistry, Wroclaw Medical University, 50-368 Wroclaw, Poland; lukasz.lewandowski@umw.edu.pl; 3 Chair and Clinical Department of Gynecology, Obstetrics and Gynecological Oncology, Medical University of Silesia, 40-211 Katowice, Poland; jstaniczek@sum.edu.pl; 4 Division of Scientific Research and Innovation in Emergency Medical Service, Department of Emergency Medical Service, Faculty of Nursing and Midwifery, Wroclaw Medical University, 51-618 Wroclaw, Poland; michal.czapla@umw.edu.pl; 5 Group of Research in Care (GRUPAC), Faculty of Health Sciences, University of La Rioja, 26006 Logroño, Spain; 6 Nursing Care and Education Research Group (GRIECE), Department of Nursing, University of Valencia, 46010 Valencia, Spain

**Keywords:** Emergency Medical Services, labor, obstetric, out-of-hospital birth, newborn

## Abstract

**Background/Objectives**: Pre-hospital delivery is an unpredictable event posing significant challenges for Emergency Medical Services (EMS) teams. Despite advances in perinatal care, emergency deliveries outside the hospital environment remain associated with increased maternal and neonatal risks. This study aimed to identify predictors of out-of-hospital delivery in EMS-attended labor cases and determinants of neonatal condition immediately after delivery. **Methods**: We conducted a retrospective analysis of 5097 EMS records of laboring women in Poland from August 2021 to January 2022, of which 2927 were included in the final study sample. Multivariate logistic regression models with multiple imputation for missing data were used to identify predictors of pre-hospital delivery and adverse neonatal condition (Apgar ≤ 7) in EMS-managed childbirths. **Results**: Pre-hospital delivery was strongly associated with second-stage labor (OR ≈ 535; *p* < 0.0001), ruptured membranes (OR ≈ 8.7; *p* < 0.0001), and fewer previous pregnancies (OR = 0.86; *p* = 0.018), and showed a trend with higher maternal heart rate (OR = 1.015; *p* = 0.083). Neonatal status classified as Apgar ≤ 7 was significantly associated with preterm birth (*p* < 0.0001), absence of fetal movements (OR ≈ 26.4; *p* = 0.025), and complications during pregnancy (*p* = 0.036). Complications during labor and lack of prenatal care were not significantly associated with increased risk of pre-hospital delivery in the model. **Conclusions**: Rupture of membranes, second-stage labor, and fewer previous pregnancies are significant predictors of pre-hospital delivery in EMS-managed cases. Absence of fetal movements and preterm gestation predict worse neonatal outcomes (Apgar ≤ 7). Early identification of these factors may enhance prehospital perinatal care and improve maternal and neonatal prognosis.

## 1. Introduction

Perinatal care is a holistic system of medical support and assistance dedicated to women during pregnancy, delivery, and the postpartum period, encompassing various forms of healthcare services [1,2]. In Poland, perinatal care is primarily provided by physicians, either residents or specialists in obstetrics, as well as certified midwives, with hospital-based delivery being the predominant mode of childbirth. Out-of-hospital births can be classified as either planned or unplanned. In the EMS context, unplanned out-of-hospital delivery most often refers to delivery occurring at the patient’s home prior to hospital arrival, rather than during transport [3]. To ensure the safety of planned home births, strict qualification criteria must be applied, limiting eligibility to patients with low-risk pregnancies. Such deliveries require the presence of qualified healthcare professionals and the availability of rapid hospital transport should complications arise [4,5]. In contrast, in unplanned emergency deliveries, the lack of clearly defined clinical guidelines contributes to concern and uncertainty among medical personnel [6,7,8]. This challenge is further compounded by the fact that most EMS teams in Poland are staffed by paramedics or nurses without a physician onboard, limiting the scope of immediate obstetric interventions.

The unpredictable nature of delivery and its often rapid progression may result in unplanned out-of-hospital deliveries. In perinatal medicine, several terms are used to describe delivery occurring outside of hospital settings, each carrying distinct clinical implications. The broadest of these terms, “out-of-hospital delivery,” encompasses any delivery outside a hospital setting, including both planned deliveries (e.g., home or birthing center births with midwifery supervision) and unplanned deliveries. Within this spectrum, the term “birth before arrival” (BBA) denotes an unintentional delivery that occurs at home or during transit to a medical facility, typically in the absence of planned medical support. A related term, “pre-hospital delivery,” is commonly used in the context of Emergency Medical Services (EMS) and refers specifically to an unplanned delivery managed by EMS personnel before reaching a labor ward. Both BBA and pre-hospital delivery underscore the unexpected and emergent nature of such events and stand in contrast to planned out-of-hospital delivery attended by skilled providers. If a laboring patient is transported by EMS and delivery occurs only after arrival at the hospital, such an event is referred to as an “in-hospital delivery following EMS transport”. This designation emphasizes that although EMS provided critical pre-hospital care and rapid transport, the birth took place under hospital care, distinguishing it from pre-hospital deliveries [3,9,10,11,12].

The absence of prenatal care, multiparity, and a history of preterm delivery are among the primary factors contributing to unplanned out-of-hospital deliveries in developed countries [13]. Despite advancements in perinatal medicine, instances still occur where delivery takes place before arrival at a labor ward. Such deliveries are frequently associated with elevated rates of maternal and neonatal perinatal morbidity and mortality [14]. Studies indicate that in the absence of prior knowledge of individual risk factors, healthcare professionals are often unable to identify patients at risk for unplanned deliveries, which may complicate intrapartum care. Therefore, understanding the factors influencing the course of such delivery, as well as neonatal outcomes, is of critical importance [15,16,17].

Pre-hospital deliveries pose a unique challenge for EMS teams [18,19]. In emergency situations, EMS personnel must respond rapidly and effectively to ensure the safe progression of labor and minimize the risk of complications for both the mother and the newborn. Research on pre-hospital deliveries in the context of EMS practice is essential for improving the quality of emergency obstetric care. Studies have shown that any physiological labor can unpredictably progress into a complicated delivery, posing significant risks to both mother and child [20]. The lack of appropriate sanitary conditions, limited access to advanced medical equipment, and the urgent nature of the situation are among the key challenges faced by EMS teams. Pre-hospital deliveries are relatively rare in the daily practice of EMS personnel, which may result in difficulties in maintaining adequate standards of care and up-to-date clinical knowledge in this domain. In many cases, the speed and effectiveness of medical intervention are crucial determinants of clinical outcomes for both the mother and the newborn during delivery [21,22,23].

Given the rarity and unpredictability of pre-hospital deliveries, especially those occurring prior to EMS arrival at a labor ward, there remains a significant gap in the literature regarding their risk factors and outcomes. The limited exposure of EMS personnel to obstetric emergencies, combined with the lack of standardized protocols, underscores the need for a better understanding of the clinical and logistical determinants of such events. Identifying these predictors is essential for optimizing EMS preparedness and improving both maternal and neonatal outcomes in the pre-hospital setting.

Considering the limited time and resources available to EMS teams during emergency calls, the ability to anticipate the likelihood of a pre-hospital delivery based on patient-related factors is of critical importance [24,25]. Identifying specific clinical predictors can enable EMS personnel to assess risk more accurately and prepare accordingly. Moreover, understanding how these predictors correlate with neonatal outcomes may improve decision-making and resource allocation during transport and initial care.

The primary objective of this study is to identify predictors of out-of-hospital delivery occurring in the presence of EMS, but prior to arrival at a labor ward, in patients who requested EMS assistance due to labor onset. The secondary objective is to determine predictors of neonatal condition immediately after such pre-hospital deliveries.

## 2. Materials and Methods

### 2.1. Study Design and Setting

A retrospective analysis was performed on the EMS records of patients with delivery out-of-hospitals in Poland from August 2021 to January 2022. The assessment was carried out using the information on medical rescue procedure cards routinely administered by EMS. The dataset included all documented cases of delivery with International Classification of Diseases (ICD-10) code: O60—preterm labor and delivery; O62.3—precipitate labor; O80—full-term, uncomplicated delivery.

### 2.2. Study Population and Data

We analyzed 5097 EMS records, of which 2095 (41%) were excluded because the patients were in the third or fourth stage of labor at the time of EMS arrival. Further analysis was conducted on 3002 cases (59%) that documented patients in the first or second stage of labor upon EMS arrival. Among these, 75 records were excluded due to missing data on the medical rescue procedure cards, resulting in a final sample of 2927 cases.

Inclusion criteria were women aged 16 years or older, who called EMS due to the onset of labor and were in the first or second stage of labor at the time of EMS intervention. Exclusion criteria included age under 16 years, being in the third or fourth stage of labor at the time of EMS arrival, when delivery had already occurred or was imminent at initial assessment and incomplete or missing data in the EMS documentation. These cases were excluded to ensure that the study population consisted only of women at risk of pre-hospital delivery, allowing valid modeling of predictors of delivery occurring prior to hospital arrival. We analyzed factors associated with pre-hospital deliveries that occurred in the presence of EMS teams. The following variables were assessed: the location and reason for EMS activation, vital signs (heart rate, blood pressure, oxygen saturation, blood glucose levels), presence of gestational diabetes, gestational hypertension, and COVID-19 infection and other comorbidities such as thrombosis, thyroid disease, depression and epilepsy. Obstetric history was also examined, including the number of pregnancies and deliveries, gestational age, antenatal course, and access to prenatal care. Additionally, we analyzed factors such as uterine contractions, rupture of membranes, stage of labor, complications during pregnancy such as the risk of preterm delivery, cervical insufficiency, fetal growth restriction and oligohydramnios, intrapartum complications, and neonatal status. Fetal movement was assessed based on maternal self-report obtained during routine EMS evaluation and documented in the medical rescue procedure card as normal, reduced, or absent. The neonatal condition of the newborn was assessed using the Apgar score. A score of 0–3 represents severe distress, 4–7 indicates moderate distress, and a score of 8–10 predicts an absence of difficulty in adjusting to extrauterine life, which is considered a normal result [26]. Intrapartum complications included hemorrhage, prolapse of the umbilical cord, retention of the placenta and eclampsia. STROBE (Strengthening the Reporting of Observational Studies in Epidemiology) guidelines were followed.

### 2.3. Statistical Analysis

#### Data Preprocessing and Statistical Analysis

All analyses were conducted in R (version 4.3.0). The analytical pipeline was designed to prioritize reproducibility, mitigate information loss due to missingness, and ensure robustness in predictive performance. An identical methodological framework was independently applied to two prediction tasks: (1) identifying predictors of successful pre-hospital delivery, and (2) predicting adverse delivery outcomes conditional on successful pre-hospital delivery. Raw data were imported from a structured .csv file. Observations containing implausible or invalid entries—such as gestational age equal to zero or non-binary parity values (multipara ∉ {0,1})—were excluded from analysis. Administrative fields lacking analytical relevance (e.g., timestamps, operational codes) were removed prior to statistical processing. Initial univariate comparisons were conducted using the Mann–Whitney U test for continuous variables (reporting medians and interquartile ranges: Q1–Q3), and either the chi-square test (with Yates’ continuity correction where appropriate) or Fisher’s exact test for categorical variables, depending on cell counts.

**Figure 1:**
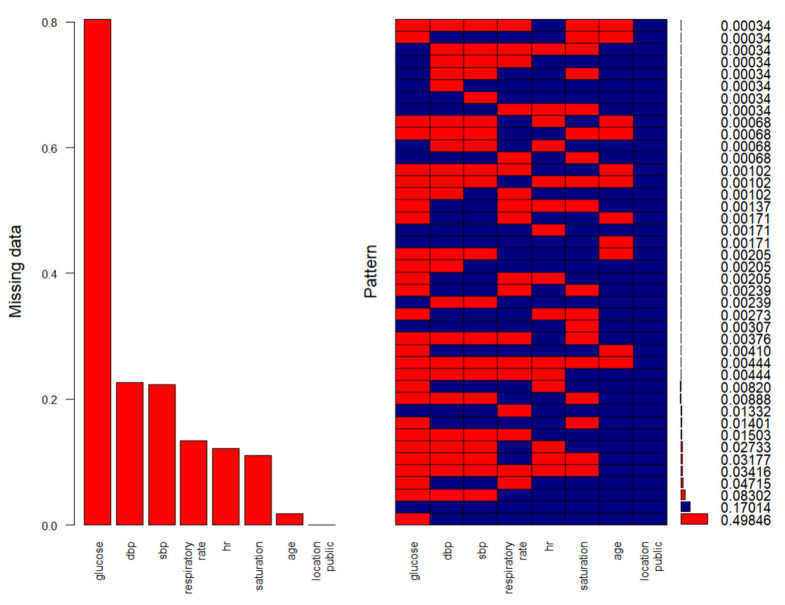
Features with missing data, and their patterns in the data before the multiple imputation (blue= observed data, red = missing).

**Figure 2:**
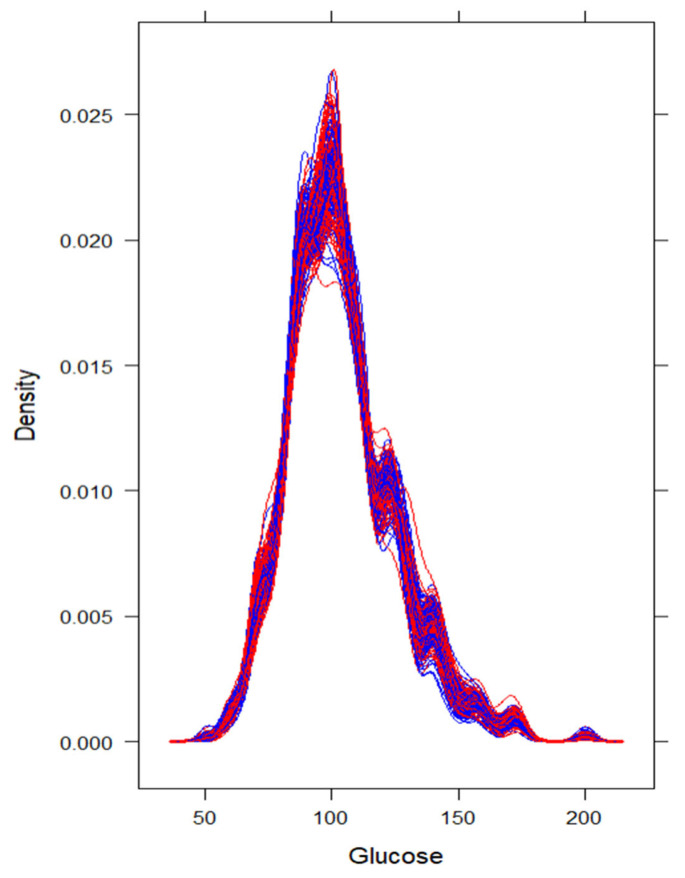
Density plot, showing the distribution of glucose concentration before and after multiple imputation of missing values (blue vs. red). Legend: blue= observed data, red = missing.

To avoid bias and loss of statistical power due to listwise deletion, we implemented Multiple Imputation by Chained Equations (MICE). Missing data were primarily observed in physiological variables, with missingness rates ranging from 1.8% (age) to 80.4% (glucose), as shown in Figure 1. Although glucose exhibited substantial missingness, its distributional characteristics were preserved after imputation (Figure 2), justifying its retention in the dataset. However, glucose was not selected into the final predictive model in the majority of imputations; therefore, no additional sensitivity analyses were conducted for its exclusion.

Two hundred imputed datasets were generated. Predictive mean matching was used for continuous variables to preserve observed distributions and reduce the risk of implausible imputations. Outcome variables, identifiers, and other non-predictive fields were excluded from both the imputation process and predictor matrix. The predictor matrix was curated using clinical domain knowledge to prevent information leakage. Diagonal entries were zeroed to avoid self-prediction.

Imputation diagnostics included graphical assessment of missingness patterns and distributional comparisons of imputed versus observed values. Continuous predictors were median-centered within each imputed dataset to aid interpretability and improve model convergence. Categorical variables were encoded as factors, and all predictors were pre-specified based on prior literature and clinical relevance, spanning demographic, physiological, obstetric, and pregnancy history domains.

For variable selection, we applied L1-regularized logistic regression (LASSO) to each imputed dataset using 10-fold cross-validation. Variables selected in ≥60% (i.e., ≥120 out of 200) of imputations were considered stably selected, balancing parsimony and model stability.

A final logistic regression model was then estimated within each imputed dataset using only the stably selected predictors. Model coefficients and standard errors were pooled across imputations using Rubin’s rules to obtain aggregate estimates and confidence intervals. To evaluate model performance, 10-fold cross-validation was conducted independently within each imputed dataset. Area under the receiver operating characteristic curve (AUC) and Brier Score were computed for each fold. Final performance metrics were averaged across imputations and folds, with associated standard deviations reported.

Clinical utility was examined using decision curve analysis (DCA) applied to a randomly selected subset of 10 imputed datasets. Net benefit curves across a range of threshold probabilities were compared against “treat-all” and “treat-none” strategies. DCA results were consistent across imputations, indicating robustness of clinical applicability.

### 2.4. Use of Artificial Intelligence Tools

Generative AI was used to assist in translating the manuscript into English, utilizing OpenAI’s ChatGPT 5. Its use was limited exclusively to language editing and translation, and the authors take full responsibility for the content of the manuscript.

## 3. Results

### 3.1. Univariate Characteristics in Context of Successful Pre-Hospital Delivery, and Less-than-Normal Delivery According to the APGAR Score—Before Applying Multiple Imputation

Patients who successfully delivered before hospitalization were older (median 30 vs. 28, *p* = 0.0114), of higher HR (median: 92 vs. 90, *p* = 0.0081), and more frequently were multiparous (80.23% vs. 69.69%, *p* = 0.0004), at the second stage of labor (95.74% vs. 3.41%, *p* < 0.0001), showing rupture of membranes (94.96% vs. 46.61%, *p* < 0.0001), and showing no complications during labor (92.64% vs. 99.51%, *p* < 0.0001).

Among pre-hospital delivery less than normal neonatal status was associated with: lower gestational week (median: 36 vs. 39, *p* = 0.0002), presence of complications during pregnancy (33.33% vs. 14.77%, *p* = 0.0360), and lack of fetal movement (0.42% vs. 9.52%, *p* = 0.0181). Descriptive statistics are shown in Table 1 and Table 2.

### 3.2. Multivariate, Post Multiple-Imputation Modeling with Pooled Estimates Subsection

#### Predicting Successful Pre-Hospital Delivery

We developed a multivariable logistic regression model (Table 3) to predict the odds of successful delivery, using multiply imputed data (m = 200) to ensure stability of estimates. Continuous variables were centered at their median values to enhance interpretability. For instance, heart rate was centered at 90 beats per minute, and number of pregnancies at 2. The model’s intercept was estimated at –5.56 (SE = 0.51, *p* < 0.001), which corresponds to baseline odds of success of approximately 0.004 (exp(–5.56)) when all predictors are at reference levels, including no complications during labor, rupture of membranes, and first stage of labor.

Conversely, presence of intact membranes was associated with a substantial decrease in odds (OR = 0.12, *p* < 0.001). Number of pregnancies, modeled as a centered continuous variable, showed a modest but statistically significant negative association (OR = 0.86 per additional pregnancy beyond the median of 2, *p* = 0.018), indicating a small reduction in odds with increasing parity.

Heart rate had a borderline positive association with outcome (OR = 1.015 per additional beat above 90 bpm, *p* = 0.083), whereas complications during labor and medical care during pregnancy did not reach statistical significance (*p* = 0.24 and *p* = 0.068, respectively).

The model demonstrated excellent predictive performance, with a 10-fold cross-validated area under the ROC curve (AUC) of 0.9767 (±0.0151), indicating high discrimination, and a mean Brier score of 0.0228 (±0.0059), suggesting good calibration.

**Figure 3:**
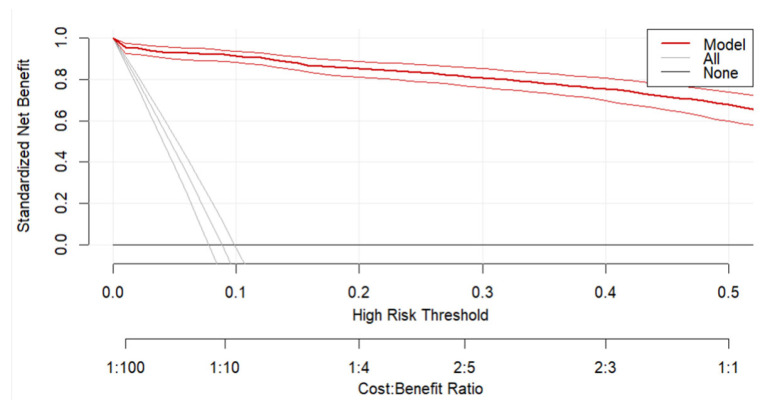
Decision Curve Analysis (DCA) evaluating the clinical utility of the predictive model (predicting successful pre-hospital deliveries). The plot displays the standardized net benefit across a range of high-risk thresholds (0 to 0.5), with the cost–benefit ratio indicated on the secondary *x*-axis. The red curve represents the net benefit of the predictive model, while the grey lines correspond to two default strategies: treating all patients (All) and treating none (None). The red lines represent the 95% Confidence Intervals (CIs) around the main red line.

The model demonstrates a consistently higher net benefit compared to both default strategies across a wide spectrum of clinically relevant threshold probabilities, indicating that using the model to guide decision-making yields greater clinical value than indiscriminately treating all or no patients. Notably, the net benefit declines as the threshold increases, reflecting the increasing trade-off between false positives and missed cases at higher risk thresholds. Confidence intervals around the model’s net benefit (thin red lines) suggest robustness of the findings. The DCA plot (Figure 3) supports the model’s potential to improve clinical decision-making by optimizing the balance between benefit and harm across plausible risk thresholds.

### 3.3. Predicting Delivery APGAR Score ≤ 7 Among All Successful Pre-Hospital Delivery

We constructed a logistic regression model to predict the odds of a less than normal delivery outcome according to the APGAR scale, centering continuous variables at their median values for interpretability (Table 4). Gestational week was centered at 39 weeks. The intercept was estimated at −3.27 (SE = 0.36, *p* < 0.001), corresponding to baseline odds of approximately 0.038 (exp(−3.27)) for ≤7 APGAR score when gestational week is at its median and there is no reported lack of fetal movement.

Among the predictors, gestational week was significantly associated with the outcome; each additional week beyond the median decreased the odds of a less than normal APGAR score by 80% (OR = 0.56, 95% CI [0.42–0.73], *p* < 0.001), highlighting the protective effect of reaching full term. On the other hand, reported lack of fetal movement was associated with a more than 26-fold increase in the odds of APGAR score ≤ 7 (OR = 26.35, 95% CI [1.52–456], *p* = 0.025), although with wide confidence intervals reflecting variability and lower event counts.

Model performance assessed via 10-fold cross-validation demonstrated moderate discrimination with an AUC of 0.78 (±0.17) and acceptable calibration with a Brier score of 0.057 (±0.024), indicating a reasonable predictive ability for clinical use but also room for improvement.

**Figure 4:**
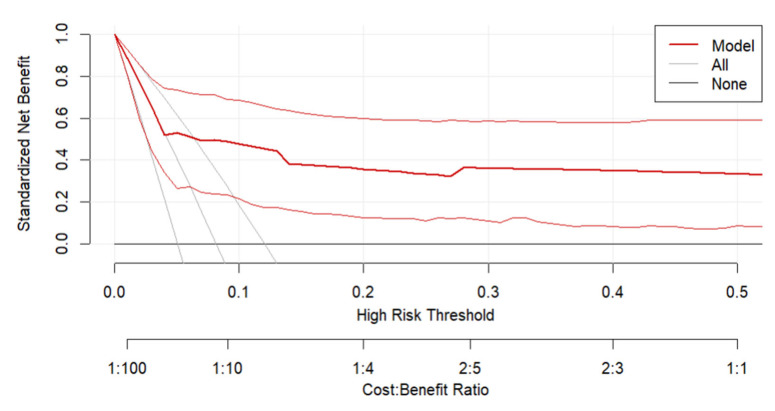
Decision Curve Analysis (DCA) evaluating the clinical utility of the predictive model (predicting less than normal neonatal status according to the APGAR scale given successful pre-hospital delivery). The plot displays the standardized net benefit across a range of high-risk thresholds (0 to 0.5), with the cost–benefit ratio indicated on the secondary *x*-axis. The red curve represents the net benefit of the predictive model, while the grey lines correspond to two default strategies: treating all patients (All) and treating none (None). The red lines represent the 95% Confidence Intervals (CIs) around the main red line.

While the model shows higher net benefit than both default strategies across lower risk thresholds—surpassing them even at the lower bound of the confidence interval below the 0.1 risk threshold. The overall width of the confidence bands remains substantial across the entire range of thresholds (Figure 4). This broad uncertainty undermines the precision of the net benefit estimates and tempers confidence in the model’s clinical utility as-is. These findings underscore the need for refinement and validation using larger, more representative cohorts before deployment in practice can be justified.

## 4. Discussion

This study demonstrated that pre-hospital delivery was most strongly associated with rupture of membranes, advancement to the second stage of labor, and a lower number of previous pregnancies. The very high odds ratio associated with second-stage labor reflects a near-deterministic clinical situation that is expected. However, in the EMS setting, this finding remains highly relevant, as decisions regarding attempted transport versus anticipated on-scene delivery must often be made under time pressure and suboptimal conditions. Furthermore, Apgar score ≤ 7 was significantly predicted by absence of fetal movements and prematurity. These findings emphasize the need for early recognition of risk factors during EMS interventions to improve perinatal outcomes.

Unplanned out-of-hospital delivery is a time-sensitive emergency that poses clinical and organizational challenges for EMS teams. While advances in perinatal care have reduced many obstetric complications, deliveries occurring before hospital arrival remain associated with increased morbidity and mortality for both mother and child [27]. Our data confirm that the majority of patients delivering before arrival were multiparous (80.23%), a pattern consistently reported in previous studies [23,27,28]. In a broader EMS context, a large US-based study showed that although obstetric emergencies constitute only 0.6% of all EMS calls, a substantial proportion of these result in pre-hospital delivery [29]. Multiparity shortens the first stage of labor, accelerating cervical dilation and increasing the risk of rapid delivery. The mean age of our study population (approx. 30 years) also reflects global trends observed among women experiencing unplanned out-of-hospital delivery [23,27,28]. Although most pre-hospital deliveries in our study occurred at term, prematurity remains a well-documented risk factor for adverse neonatal outcomes. French national data have shown that patients delivering outside maternity wards had an increased likelihood of giving delivery before 37 weeks’ gestation [30]. In our sample, most pre-hospital deliveries occurred around 39 weeks of gestation, and only 7.75% occurred in public locations, suggesting that, in the Polish context, these events predominantly take place in private homes and often involve term pregnancies.

Early intervention in such cases is critical, as timely transport to perinatal centers with neonatal intensive care units can improve outcomes [31]. Similar conclusions were reached by Sumikawa et al., who found that anticipated preterm labor was the most common reason for transporting pregnant women in Japan [32]. Moreover, data from Polish HEMS services have also shown that premature labor (ICD-10: O60) is the most frequently reported obstetric emergency during EMS dispatches [33]. Contrary to some previous studies, our multivariate model found that a higher number of previous pregnancies decreased the odds of pre-hospital delivery by approximately 16% per additional pregnancy. This partially contradicts results by Rzońca et al., who observed that a higher number of deliveries increased the risk of preterm labor [28]. This discrepancy may stem from differing definitions (preterm vs. pre-hospital delivery) or the influence of other interacting factors such as maternal age and parity structure. Among neonatal risk factors, gestational age and fetal movement stood out, with longer gestational duration being associated with a lower probability of an Apgar score ≤ 7, consistent with previous evidence linking prematurity to poorer neonatal outcomes [23,27]. Furthermore, newborns with low Apgar scores were significantly more often born to mothers who experienced complications during pregnancy, suggesting that antenatal morbidity may contribute to adverse neonatal condition at birth. Strikingly, absence of fetal movement was associated with substantially increased odds of neonatal compromise, making it one of the strongest warning signs available to EMS personnel.

This finding supports the inclusion of fetal movement assessment in prehospital obstetric protocols. Vital sign monitoring remains essential during obstetric EMS calls. In Poland, pulse oximetry and blood pressure measurement are the most frequently performed interventions during care for laboring women [20]. Rupture of membranes emerged as one of the clearest predictors of imminent delivery. In our analysis, its presence markedly increased the likelihood of pre-hospital delivery. This is consistent with previous EMS research, where ruptured membranes were reported in up to 70% of patients in active labor, and often served as the direct reason for EMS intervention [34]. The most dominant factor, however, was labor stage. Patients already in the second stage at the time of EMS assessment were dramatically more likely to deliver before arrival compared to those in the first stage. Given that this stage can last less than 30 min in multiparas [35], rapid progression often precludes hospital transfer and requires EMS readiness for delivery in the field. Altogether, our findings reinforce the utility of several accessible indicators membrane status, labor stage, fetal movement, maternal heart rate, for predicting both the likelihood of pre-hospital delivery and neonatal risk. While some of these signs are already used informally in EMS practice, our study provides statistical evidence supporting their predictive value. Integration of these markers into formal EMS protocols may improve prehospital care and support more informed decision-making regarding whether immediate transport is feasible or whether delivery should be anticipated and managed on scene during obstetric emergencies [12,36,37]. Qualitative research conducted in Iran emphasized that barriers such as limited EMS training, lack of standardized protocols, and cultural constraints-especially the discomfort with male personnel attending delivery in conservative communities can significantly undermine the quality of prehospital obstetric care [3]. This highlights the need for context-sensitive training and clear guidelines to ensure safe and respectful maternal care across various healthcare systems. Building on this, we propose that EMS protocols incorporate simple obstetric indicators such as rupture of membranes, labor stage, and fetal movements into structured risk assessment tools. Such integration may enhance EMS readiness and ultimately improve maternal and neonatal outcomes in unplanned out-of-hospital deliveries. From a practical perspective, these findings support the need for EMS decision-making to be based on real-time clinical assessment rather than assumptions about prior healthcare contacts, particularly in the absence of standardized prehospital obstetric triage pathways. In healthcare systems where women are advised to contact hospital labor wards prior to admission, such advice may influence the timing of presentation and indirectly affect the risk of pre-hospital delivery; however, these processes are not standardized or captured in Polish EMS data.

### Study Limitation

This study has several limitations that should be acknowledged. Firstly, the retrospective design of the study relies on the accuracy and completeness of EMS documentation, which may introduce information bias, including the lack of systematically recorded data on the duration of labor prior to EMS arrival and on any prior contacts with hospital services before calling EMS. Secondly, some cases were excluded due to missing or incomplete data, potentially affecting the representativeness of the sample and limiting the generalizability of the findings. Thirdly, EMS personnel did not employ standardized obstetric assessment tools, which might have influenced the precision and uniformity of clinical evaluations. Another limitation of this study is the aggregated nature of the variables “complications during pregnancy” and “complications during labour”. These categories encompassed a heterogeneous range of conditions documented in EMS records, each with potentially different prognostic significance. While this approach reflects the realities of pre-hospital documentation and limited event counts for individual conditions, it restricts the clinical interpretability of their specific effects within the predictive models. Moreover, EMS documentation typically lacks information regarding the prescribed medications and dosages taken by patients, limiting the ability to account for pharmacological factors that might influence maternal or neonatal outcomes. Moreover, the study was conducted solely in Poland, and differences in EMS systems and perinatal care protocols internationally may restrict the applicability of these results to other settings. Finally, the analysis was limited to immediate neonatal outcomes assessed via the Apgar score, without long-term follow-up of maternal or neonatal health, which could provide additional insight into the clinical significance of the identified predictors.

## 5. Conclusions

This study identified several key predictors of pre-hospital delivery among women attended by EMS teams. The likelihood of birth occurring before hospital arrival was significantly associated with the presence of amniotic fluid rupture and advancement to the second stage of labor at the time of EMS assessment. Additionally, a lower number of previous pregnancies was linked to an increased probability of pre-hospital delivery. Regarding neonatal outcomes, a lower gestational age and the absence of fetal movements were significant predictors of a neonatal status rated as less than normal (≤7) according to the Apgar score. Specifically, the risk of compromised neonatal condition decreased markedly with increasing gestational age. These findings highlight the importance of careful evaluation of simple clinical indicators available during EMS interventions, which may improve the ability to predict both imminent pre-hospital deliveries and potential risks to neonatal health.

## Figures and Tables

**Table 1:** Population sample characteristics (before multiple imputation) in context of pre-hospital delivery.

Feature (Quantitative)	Lack of Pre-Hospital Delivery (Total N = 2669)							Pre-Hospital Delivery (Total N = 258)							*p*
	N	% Total N	Median	Min	Max	Q1	Q3	N	% Total N	Median	Min	Max	Q1	Q3	
age	2628	98.4638	28	15	46	23	34	246	95.3488	30	16	44	25	34	0.0114
respiratory rate	2331	87.3361	16	12	20	14	18	204	79.0698	16	12	20	14	18	0.8627
saturation	2385	89.3593	98	90	100	98	99	218	84.4961	98	93	100	97	99	0.3823
sbp	2088	78.2315	130	70	180	120	140	186	72.0930	130	90	180	120	140	0.7389
dbp	2081	77.9693	80	40	130	70	85	184	71.3178	80	51	108	79	84	0.4535
hr	2354	88.1978	90	56	160	81	100	218	84.4961	92	60	160	85	104	0.0081
glucose	520	19.4830	101	51	200	90	116	53	20.5426	104	68	173	87	126	0.5733
gestational week	2669	100.0000	39	23	42	37	40	258	100.0000	39	23	42	38	40	0.0839
num pregnancies	2669	100.0000	2	1	11	1	4	258	100.0000	3	1	12	2	4	0.0020
num labors	2669	100.0000	2	1	11	1	3	258	100.0000	2	1	11	2	3	0.0018
Feature (qualitative)	Lack of pre-hospital delivery		Pre-hospital delivery		*p*										
	N	%	N	%											
location public: yes	168	6.29%	20	7.75%	0.3619										
COVID-positive: no	1504	56.35%	130	50.39%	0.3870										
COVID-positive: yes	66	2.47%	3	1.16%											
COVID-positive: missing data	1099	41.18%	125	48.45%											
multipara: yes	1860	69.69%	207	80.23%	**0.0004**										
complications during pregnancy: yes	473	17.72%	42	16.28%	0.5611										
medical care during pregnancy: yes	2529	94.75%	240	93.02%	0.2399										
stage of labor: 1	2578	96.59%	11	4.26%	**<0.0001**										
stage of labor: 2	91	3.41%	247	95.74%											
bleeding: yes	237	8.88%	16	6.20%	0.1478										
reduced fetal movement: yes	13	0.49%	0	0.00%	0.5266										
lack of fetal movement: yes	45	1.69%	3	1.16%	0.7075										
Rupture of membranes: no	1425	53.39%	13	5.04%	**<0.0001**										
comorbidities: yes	315	11.80%	28	10.85%	0.6507										
gestational diabetes: yes	142	5.32%	11	4.26%	0.4665										
gestational hypertension: yes	52	1.95%	8	3.10%	0.2122										
complications during labor: yes	13	0.49%	19	7.36%	**<0.0001**										
neonatal status: normal	0	0.00%	237	91.86%	-										
neonatal status: requires attention	0	0.00%	12	4.65%											
neonatal status: needs life-saving	0	0.00%	4	1.55%											
neonatal status: stillbirth	0	0.00%	5	1.94%											

**Table 2:** Population sample characteristics (before multiple imputation) in context of less than normal (Apgar score ≤ 7) neonatal status given pre-hospital delivery.

Feature (Quantitative)	Apgar Score ≥ 8 Pre-Hospital Delivery (Total N = 237)							Apgar Score ≤ 7 Pre-Hospital Delivery (Total N = 21)							*p*
	N	% Total N	Median	Min	Max	Q1	Q3	N	% Total N	Median	Min	Max	Q1	Q3	
age	226	95.3586	30	16	44	25	34	20	95.2381	31	21	40	26	35	0.4685
respiratory rate	190	80.1688	16	12	20	14	18	14	66.6667	16	12	20	13	18	0.7646
saturation	201	84.8101	98	93	100	97	99	17	80.9524	98	96	100	98	99	0.2613
sbp	173	72.9958	130	90	180	120	140	13	61.9048	130	96	156	120	140	0.8700
dbp	171	72.1519	80	51	108	75	84	13	61.9048	80	65	90	80	84	0.5930
hr	203	85.6540	91	60	160	85	105	15	71.4286	100	70	157	90	104	0.2979
glucose	49	20.6751	104	68	164	87	121	4	19.0476	114	93	173	97	150	0.3867
gestational week	237	100.0000	39	35	42	38	40	21	100.0000	36	23	41	33	39	0.0002
num pregnancies	237	100.0000	3	1	12	2	4	21	100.0000	2	1	7	2	4	0.9855
num labors	237	100.0000	2	1	11	2	3	21	100.0000	2	1	7	2	3	0.9372
Feature (qualitative)	Apgar score ≥ 8 pre-hospital delivery		Apgar score ≤ 7 pre-hospital delivery		Fisher’s *p*										
	N	%	N	%											
location public: yes	18	7.59%	2	9.52%	0.5000										
COVID-positive: no	118	49.79%	12	57.14%	0.7513										
COVID-positive: yes	3	1.27%	0	0.00%											
COVID-positive: missing data	116	48.95%	9	42.86%											
multipara: yes	191	80.59%	16	76.19%	0.4028										
complications during pregnancy: yes	35	14.77%	7	33.33%	**0.0360**										
medical care during pregnancy: yes	221	93.25%	19	90.48%	0.4420										
stage of labor: 1	9	3.80%	2	9.52%	0.2223										
stage of labor: 2	228	96.20%	19	90.48%											
bleeding: yes	15	6.33%	1	4.76%	0.6189										
reduced fetal movement: yes	0	0.00%	0	0.00%	-										
lack of fetal movement: yes	1	0.42%	2	9.52%	**0.0181**										
Rupture of membranes: no	10	4.22%	3	14.29%	0.0782										
comorbidities: yes	26	10.97%	2	9.52%	0.5955										
gestational diabetes: yes	10	4.22%	1	4.76%	0.6146										
gestational hypertension: yes	7	2.95%	1	4.76%	0.4979										
complications during labor: yes	19	8.02%	0	0.00%	0.1873										
neonatal status: normal	237	100.00%	0	0.00%	-										
neonatal status: requires attention	0	0.00%	12	57.14%											
neonatal status: needs life-saving	0	0.00%	4	19.05%											
neonatal status: stillbirth	0	0.00%	5	23.81%											

**Table 3:** The developed model aimed at predicting pre-hospital births.

Feature	Interpretation	β	β SE	t	df	*p*	OR	OR −95% CI	OR 95% CI
(Intercept)	Baseline odds of pre-hospital delivery given, rupture od membranes, no complications during labor, HR = 90 bpm, no medical care during pregnancy, one past pregnancy, and first stage of labor	−5.5587	0.5135	−10.8258	2908.9340	<0.0001	0.0039	0.0014	0.0105
rupture of membranes: yes vs no	Fold difference in baseline odds between patients with intact membranes vs. rupture of membranes	−2.1573	0.3565	−6.0506	2915.9180	<0.0001	0.1156	0.0575	0.2326
complications during labor: yes	Fold difference in baseline odds between patients with complications during labor vs. no complications	0.8653	0.7427	1.1650	2912.8240	0.2441	2.3757	0.5541	10.1869
HR (median–centered)	Fold difference in baseline odds with each 1 bpm increase in HR	0.0149	0.0086	1.7326	2115.4620	0.0833	1.0150	0.9980	1.0323
medical care during pregnancy: yes vs no	Fold difference in baseline odds between patients with medical care during pregnancy vs. those without it	0.7607	0.4168	1.8251	2913.7360	0.0681	2.1397	0.9453	4.8432
number of pregnancies (median-centered)	Fold difference in baseline odds with each subsequent pregnancy	−0.1468	0.0622	−2.3600	2914.2590	0.0183	0.8634	0.7643	0.9754
stage of labor:2	Fold difference in baseline odds between patients at the second stage of labor vs. first	6.2818	0.3427	18.3291	2912.2240	<0.0001	534.7309	273.1524	1046.8043

**Table 4:** The developed model aimed at predicting neonatal status according to the APGAR scale ≤ 7, given successful pre-hospital delivery.

Feature	Interpretation	β	β SE	t	df	*p*	OR	OR −95% CI	OR 95% CI
(Intercept)	Baseline odds neonatal status according to the APGAR score ≤ 7 given successful pre-hospital delivery, assuming 39th gestational week and presence of any fetal movement	−3.2687	0.3550	−9.2066	252.9980	<0.0001	0.0381	0.0190	0.0763
gestational week (median- centered)	Fold change in baseline odds with each one gestational week increase	−0.5880	0.1377	−4.2709	252.9980	<0.0001	0.5554	0.4241	0.7275
lack of fetal movement: yes vs no	Fold change in baseline odds between cases of lack of fetal movement vs. any movement (normal or reduced)	3.2718	1.4553	2.2482	252.9980	0.0254	26.3578	1.5211	456.7256

## Data Availability

The datasets used and/or analysed during the current study are available from the corresponding author on reasonable request.

## Abbreviations

The following abbreviations are used in this manuscript:

AUC	Area Under the Curve
BBA	Birth Before Arrival
CI	Confidence Interval
COVID-19	Coronavirus Disease 2019
DCA	Decision Curve Analysis
EMS	Emergency Medical Services
HR	Heart Rate
ICD-10	International Classification of Diseases, 10th Revision
IQR	Interquartile Range
LASSO	Least Absolute Shrinkage and Selection Operator
LOHS	Length of Hospital Stay
MICE	Multiple Imputation by Chained Equations
OR	Odds Ratio
ROC	Receiver Operating Characteristic
SBP	Systolic Blood Pressure
SE	Standard Error
